# Calcium-containing inorganic immunity booster drives the enrichment of specific root endophytic microbiota against *Ralstonia solanacearum*

**DOI:** 10.1186/s40168-025-02228-1

**Published:** 2025-12-12

**Authors:** Jingjing Bi, Buqing Wei, Enzhao Wang, Xian Zeng, Xingyan Liu, Miaomiao Sun, Hailei Wei, Shidong Li, Mingshun Chen, Jingjing Peng, Alin Song, Keke Yi, Fenliang Fan

**Affiliations:** 1 State Key Laboratory of Efficient Utilization of Arid and Semi-Arid Arable Land in Northern China, Key Laboratory of Plant Nutrition and Fertilizer, Ministry of Agriculture and Rural Affairs, Institute of Agricultural Resources and Regional Planning, Chinese Academy of Agricultural Sciences, South Zhongguancun Street No.12, Beijing, 100081 China; 2 Institute of Plant Protection, Chinese Academy of Agricultural Sciences, Beijing, 100193 China; 3 Department of Entomology, Kansas State University, Manhattan, KS 66506 USA; 4 College of Resources and Environmental Sciences, China Agricultural University, Beijing, 100193 China

**Keywords:** Root endophytic microbe, Calcium, *Ralstonia solanacearum*, Plant immunity, Synthetic bacterial community

## Abstract

**Background:**

Engineering root microbiomes holds great promise to enhance plant health. Enhanced plant resistance via breeding or genetic modification can promote recruiting beneficial microbes, but is challenging to achieve.

**Results:**

Here we showed that calcium (Ca) addition caused significant changes in tomato physiology, resulting in enhanced immunity towards the pathogen *Ralstonia solanacearum* along with increased levels of salicylic acid (SA), sugar content, and defense enzyme activities in roots. High Ca levels significantly altered the root microbiomes, enriching sixteen bacterial genera, including *Dyella japonica*, *Rhodanobacter glycinis*, *Paenibacillus polymyxa*, and *Pseudomonas aeruginosa*, with the mostly enriched genus showing a 16.5-fold increase in the relative abundance compared to no Ca addition. Associated with the enhancement of these bacterial genera, tomato wilt incidence was reduced from 80 to 0%. The growth of these four bacterial strains was promoted to varying extent by the addition of SA, sucrose, trehalose, and Ca in vitro. All the four bacterial strains had antagonistic ability against *R. solanacearum*. A synthetic microbial community (SynCom) of these four strains significantly inhibited *R. solanacearum* growth in whole root exudates treated with Ca, and the combination of SynCom and Ca addition resulted in nearly eradication of the tomato bacterial wilt.

**Conclusions:**

Our findings revealed an effective and simple method for enriching beneficial microbiomes to protect plants against pathogens. Our results also suggested that combination of immunity boosters and beneficial microbes offers a novel strategy to combat soil-borne diseases.

Video Abstract

**Supplementary Information:**

The online version contains supplementary material available at 10.1186/s40168-025-02228-1.

## Background

Soil-borne diseases pose serious threat to global food security as they reduce the yield and quality of field crops [1, 2]. Continuous application of chemical pesticides for controlling soil-borne diseases has resulted in negative impact on biodiversity, water quality, and food safety [3, 4]. Thus, eco-friendly and safe plant protection techniques are urgently needed. Roots harbor complex microbiomes, which play vital roles in the health and development of host plants [5–7]. Exploring microbial potentials is considered as promising, eco-friendly, and safe alternate for sustainable plant disease control [8].

Various strategies, including soil amendment, microbiome modification, and plant modification [9–11], have been adopted to manipulating root microbiomes for disease control [12]. Soil amendments, such as adding organic manure and chemical fertilizer, can significantly cause changes in soil properties, and diversity and function of root microbiomes [13, 14]. Soil amendments were initially applied to improve soil fertility rather than manipulate root microbiomes. Microbiome modification, through inoculating natural or genetically modified beneficial microbes individually or as synthetic communities, results in enhanced plant ability to suppress pathogens [15–17]. Several bacterial genera, such as *Bacillus*, *Pseudomonas*, and *Paenibacillus*, are widely used as microbial inoculants [18–20]. Plant host modification involves various means to change the profiles of root exudates, which can attract beneficial microbes from surrounding to roots [21, 22]. Diverse candidate compounds from plant root exudates have been reported, including plant defense hormones salicylic acid (SA) and jasmonic acid (JA). These chemicals from root exudates can attract disease-suppressive microbes and modulate colonization of the root microbiomes [23–25]. It is expected that integrating abovementioned strategies is more effective than single strategy to maximize microbial biocontrol potential, but technical barriers and limited understanding of synergistic effects have hindered attempts to integrate these strategies effectively.

Nutrient availability is a critical factor impacting soil-borne diseases in plants [26, 27]. Some nutrients not only mediate plant physiology that directly affect plant’s ability to defend itself against pathogens, but also shape microbiome that indirectly impact plant disease resistance [28]. Calcium (Ca) is a particular representative as it is not only a structural component of cell wall and membrane, but also a second messenger that mediates signal transduction in plant defense responses to enhance plant antioxidant capacity and induce the expression of defense-related genes [29–31]. Exogenous Ca application indicated that it could promote plant growth, prime plant resistance, and induce downstream metabolic cascades supporting higher immunity [32–34]. Recent studies have also demonstrated that Ca is capable of facilitating plant to enrich beneficial microbial taxa [35], but the precise mechanisms by which Ca stimulates recruitment of beneficial microbes remain largely unknown.

Building on studies showing direct effects of Ca on plant resistance, this work explores its broader roles in root microbiome assembly for disease suppression. We hypothesize that Ca primes plant defense responses that help plants to recruit beneficial microbes that can suppress *R. solanacearum*, the causative agent of bacterial wilt affecting more than 200 crops [36]. We first planted tomato plants under seven Ca levels and monitored plant defense responses and changes in root microbiomes under these conditions with multi-omics approaches. Subsequently, we isolated the dominant root-associated bacterial taxa and constructed a synthetic bacterial community (SynCom) by testing their direct effects on suppressing *R. solanacearum*, preference to Ca, and response to root-derived metabolites associated with defense responses. Last we assessed the effectiveness of combined Ca supplementation and SynCom on disease control. We found that Ca increased plant defense responses and selected specific microbial taxa through defense-associated metabolite enrichment. The assembled SynCom reduced wilt incidence rates in tomato plants alone, and showed great synergy in suppressing tomato bacterial wilt in combination of Ca supplementation.

## Materials and methods

### Plant materials and growth conditions

The tomato variety Zhongza 301, provided by China Vegetable Seed Technology Co., Ltd. (Beijing, China), was used in this study. Tomato seeds were surface-sterilized with 0.15% KMnO_4_ for 1 h, washed three times with water, and sown directly into seedling trays containing 72 slots, each filled with 72 g of peat substrate. After 4 weeks of greenhouse cultivation, healthy seedlings at the three-leaf stage were selected and transplanted into individual plastic pots (18 cm in diameter and 19 cm in height), each containing 2 kg of soil. The experiment was conducted in a greenhouse located in Langfang City, Hebei Province, China, from 29^th^ of April to 26^th^ of June, 2021. The soil used for cultivation was collected from a barren farmland in Yueyang City, Hunan Province, China. The soil had the following physicochemical properties: pH 5.8; total N of 0.69 g kg^−1^; NH_4_^+^-N content of 4.49 mg kg^−1^; NO_3_^−^-N content of 3.61 mg kg^−1^; soil available phosphorus of 25.54 mg kg^−1^; soil available potassium of 56.1 mg kg^−1^; and soil exchangeable Ca of 11.69 cmol kg^−1^.

### Experimental design

Based on a pilot experiment that revealed the growth response curve of tomato plants to Ca addition at rates of 0, 50, 100, 200, 300, 400, 500, 600, 700, and 800 mg Ca kg^−1^ soil (Fig. S1), we established a broad Ca gradient to investigate how Ca influences plant resistance and modulates root microbiomes. The specific treatments used in this study were as follows: (1) no Ca fertilizer (Ca0), (2) 50 mg Ca kg^-1^ soil (Ca50), (3) 100 mg Ca kg^-1^ soil (Ca100), (4) 200 mg Ca kg^-1^ soil (Ca200), (5) 300 mg Ca kg^-1^ soil (Ca300), (6) 400 mg Ca kg^-1^ soil (Ca400), and (7) 800 mg Ca kg^-1^ soil (Ca800). Among these, Ca300 and Ca400 were identified as optimal concentrations for simultaneously promoting plant growth and disease control, while Ca800 represented an excessive application level that enhanced disease control but inhibited plant growth. Anhydrous calcium chloride was used as Ca fertilizer. Additionally, all treatments received a uniform basal application of nutrients at 150 mg kg^-1^ soil: N as urea, and P and K as KH_2_PO_4_. There were 12 replicates for each treatment. All fertilizers were dissolved in water and thoroughly mixed into the soil 1 week before transplanting the seedlings.

### Pathogen inoculation and disease assessment

A highly virulent strain of *R. solanacearum* named P380 [37] was used in this study. The strain was first cultured on the TZC agar medium (tryptone 10 g L^−1^, casamino acid 1 g L^−1^, glucose 5 g L^−1^, 2, 3, 5-triphenyltetrazolium chloride 0.05 g L^−1^, agar 15 g L^−1^, pH 7.0) for 48 h at 28℃. The resulting colonies were then transferred to NB liquid medium (beef extract 3 g L^−1^, glucose 10 g L^−1^, tryptone 5 g L^−1^, yeast extract 0.5 g L^−1^, pH 7.0) and incubated at 28℃ for 48 h in a rotary shaker at 180 rpm. Tomato plants were inoculated at the six-leaf stage. For inoculation, tomato roots were injured using a razor blade approximately 2 cm away from the stem base, and 50 mL of *R. solanacearum* suspension (approximately 10^9^ CFU mL^−1^, OD_600_ = 1) was immediately applied to the rhizosphere soil.

In our pot experiment, we observed that the disease symptoms first appeared as whole-plant wilting during the high-temperature periods around noon on the first day, followed by recovery to normal conditions by the next morning. This cycle of wilting and recovery occurred repeatedly before plants eventually reached a stage of permanent wilting. This symptom progression did not conform to the standard bacterial wilt severity scoring system. Consequently, we used disease incidence as the primary indicator to assess the occurrence and progression of bacterial wilt. Disease symptoms were monitored every 2 days after *R. solanacearum* inoculation, and daily observations were conducted once visible symptoms appeared. The following formula was used to calculate wilt incidence:

$$\mathrm{Disease}\;\mathrm{incidence}\;\left(\%\right)=\frac{\mathrm{Number}\;\mathrm{of}\;\mathrm{infected}\;\mathrm{plants}}{\mathrm{Total}\;\mathrm{number}\;\mathrm{of}\;\mathrm{plants}\;\mathrm{investigated}}\times100$$

### Sample collection and physicochemical analysis

**Fig. 1:**
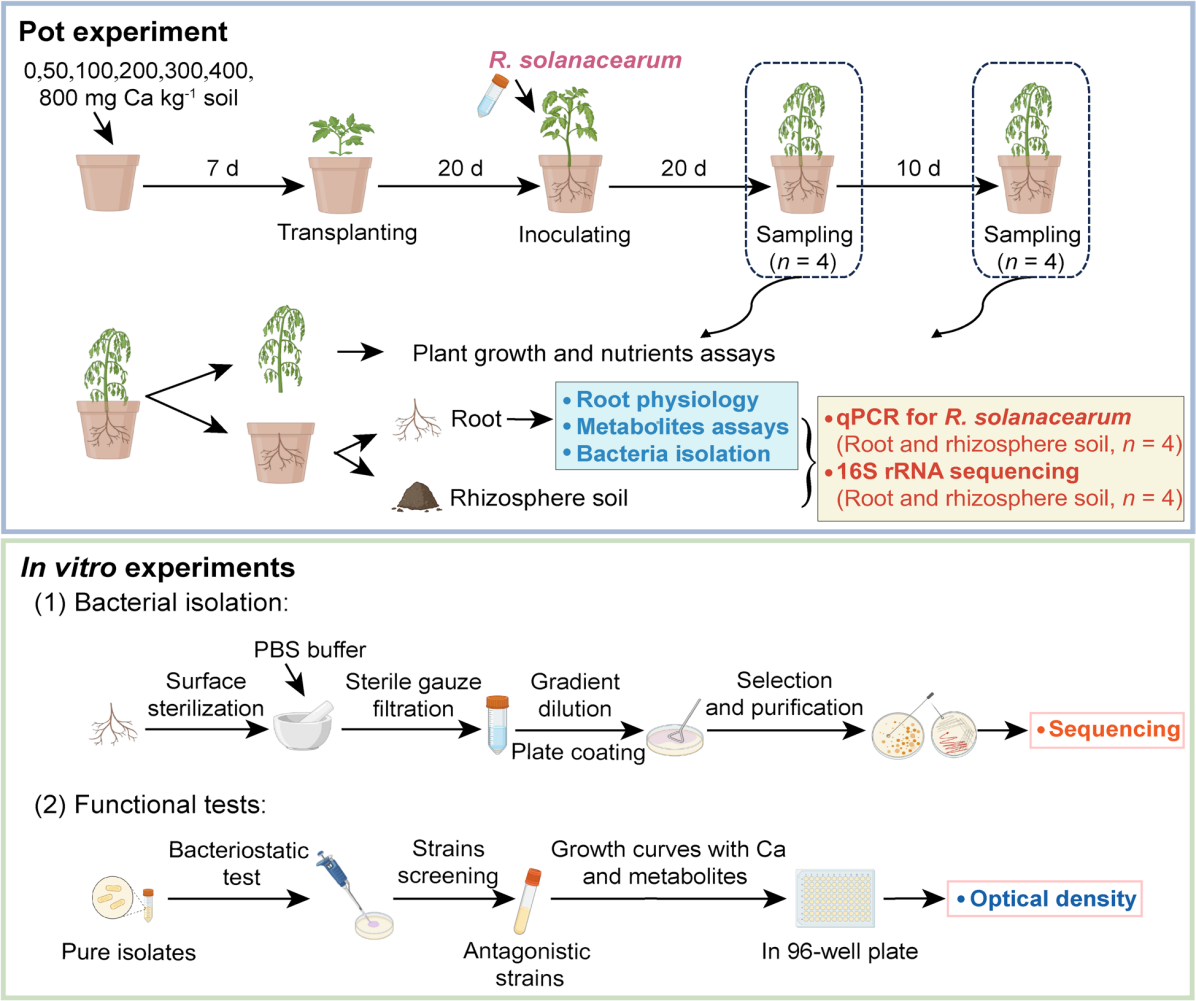
Schematic overview of the experimental design. The top part of the figure shows the procedure of pot experiments and sampling with seven Ca levels (0, 50, 100, 200, 300, 400, and 800 mg Ca kg^-1^ soil in CaCl_2_). Root and rhizosphere soil samples were collected on day 20 and day 30 after inoculation with* R.* *solanacearum*. The bottom part of the figure shows the isolation of root endophytic bacteria and the functional testing of putative beneficial isolates. All panels were created using BioRender (https://www.biorender.com)

Based on a comprehensive evaluation of disease progression dynamics and Ca-mediated protective effects, two sampling time points were selected: day 20 and day 30 after *R. solanacearum* inoculation. Day 20 represents the intermediate disease phase, characterized by established pathogen colonization and active disease progression. Day 30 corresponds to the disease stabilization phase, during which plants treated with higher Ca levels (≥ 300 mg Ca kg^-1^ soil) maintained stable resistance (Fig. 1). At each sampling time points, four representative pots were selected per treatment for analysis, while the remaining pots were kept for continued disease monitoring. Plant growth parameters, including plant height and shoot fresh weight, were measured and recorded. Shoots were cut at the base above the soil surface, heated at 105°C for 30 min, and then dried at 65°C for 24 h for subsequent total N and total Ca determination. Roots were carefully washed with water, wiped with paper tissues, wrapped in tinfoil, and immediately frozen in liquid nitrogen. Approximately 50 g of rhizosphere soil tightly attached to roots was collected and stored at − 80℃ for molecular analysis, while an additional 100 g of rhizosphere soil was stored at 4℃ for analysis of exchangeable Ca content. Total N was determined using the Kjeldahl distillation. Soil exchangeable Ca was extracted using ammonium acetate and quantified by atomic absorption spectrophotometry. Total Ca content in plant tissues was determined using an inductively coupled plasma atomic emission spectrometer (ICP-AES) after acid digestion. Soil pH was measured using the potentiometric method.

Raising antioxidant enzyme activity is a key strategy by which plants resist pathogen-induced stress. In this study, the activities of major antioxidant enzymes, including superoxide dismutase (SOD, which scavenges superoxide radicals and reduces oxidative damage), peroxidase (POD, which eliminates hydrogen peroxide and participates in plant defense responses), and catalase (CAT, which efficiently degrades hydrogen peroxide and mitigates oxidative damage), as well as the levels of malondialdehyde (MDA, a marker for oxidative stress and lipid peroxidation), were measured. All measurements were conducted using commercial assay kits obtained from Suzhou Grace Biotechnology Co., Ltd. (Suzhou, China), including the SOD Assay Kit (#G0101W), POD Assay Kit (#G0107W), CAT Assay Kit (#G0105W), and MDA Assay Kit (#G0109W). Assays were performed according to the manufacturer’s protocols.

### Root metabolome

The extraction and determination of root metabolites were conducted as follows: 100 mg of root tissues were ground in liquid nitrogen, and the resulting powder was dissolved into prechilled 80% methanol. The samples were incubated on ice for 5 min, followed by centrifugation at 15,000* g* under 4℃ for 20 min. The supernatants were then diluted to a final concentration of 53% methanol using LC-MS grade water, transferred to new Eppendorf tubes, and centrifuged again at 15,000* g* under 4℃ for 20 min. The resulting supernatants were subjected to chemical composition analysis using an ultra-high-performance liquid chromatography-tandem mass spectrometry (UHPLC-MS/MS) system (Thermo Fisher Scientific, Germany), coupled with an Orbitrap Q Exactive™ HF-X mass spectrometer (Thermo Fisher Scientific, Germany). Sample analysis was conducted by Novogene Co., Ltd. (Beijing, China) under a commercial service contract. The extraction and determination of plant hormones from root tissues were carried out following previously described methods [38].

### Root and rhizosphere microbial community

Total microbial DNA from root and rhizosphere soil samples was extracted using a Fast DNA SPIN Kit for Soil (MP Biomedical, Santa Ana, CA, USA) according to the manufacturer’s protocol. DNA quality was checked on 1.0% an agarose gel and quantified on a Nanodrop2000 spectrophotometer (Thermo Fisher Scientific, Waltham, USA). The DNA was subjected to bacterial 16S rRNA amplicon sequencing to determine the composition and diversity of bacterial communities. For sequencing, the 16S rRNA gene V3-V4 hypervariable region was amplified with the primer pair 515 F (5’-GTGCCAGCMGCCGCGGTAA-3’) and 806R (5’-GGACTACHVGGGTWTCTAAT-3’) [39], each with a unique 12-bp barcode. Amplification was carried out in 50 µL solution containing 36.5 µL ddH_2_O, 5 µL 10 × buffer, 4 µL dNTP, 0.5 µL rTaq (Takara), 1 µL of primer 515 F (10 µM) solution, 1 µL of primer 806R (10 µM) solution, and 2 µL DNA template. The PCR amplification was performed with the following condition: pre-denaturation at 94℃ for 5 min, followed by 30 cycles of denaturing at 94℃ for 45 s, annealing at 55℃ for 35 s and extension at 72℃ for 45 s, with a final extension at 72℃ for 10 min. PCR fragments were checked via electrophoresis on 1% agarose gels, and a QuantiT PicoGreen dsDNA Assay Kit (Invitrogen) was used to determine the concentrations of the PCR products. Equal quantities of PCR products were pooled and purified via agarose gels using a DNA Purification Kit (Tiangen Technologies, Beijing, China). Sequencing was performed on the Illumina Hiseq2500 PE250 platform by Novogene Co., Ltd. (Beijing, China).

Raw bacterial sequences were trimmed to 250 bp and quality-filtered using USEARCH (version 8.1.1861_win64) [40], with a maximum expected error threshold of 0.5. The remaining reads were denoised using the UNOISE3 algorithm to obtain amplicon sequence variant (ASV) table. Representative sequences were taxonomically assigned using the RDP classifier (version 2.12) against the RDP database [41]. A total of 2,809,543 raw sequences were generated from 112 samples (56 root samples and 56 rhizosphere soil samples). After quality filtering and chimera removal, 2,633,806 high-quality sequences remained, which were assigned to 4511 bacterial ASVs. Plant genomic sequences were manually removed when an ASV classified as Chloroplast and confirmed by NCBI blast. The ASV table was rarefied to 10,000 sequences per sample for the 16S rRNA gene in subsequent analyses.

### Pathogen quantification with quantitative PCR (qPCR)

To quantify the density of *R. solanacearum* in tomato roots and rhizosphere soil, qPCR was conducted using an ABI prism 7900 system (Applied Biosystems, USA). The primers specific to *R. solanacearum*, 759R (5’-GTCGCCGTCAACTCACTTTCC-3’) and 760F (5’-GTCGCCGTCAGCAATGCGGAATCG-3’), were used for amplification [42]. The amplification was carried out in 15 µL solution containing 4.2 µL ddH_2_O, 7.5 µL SYBR^®^ Premix Ex Taq, 0.3 µL ROX Reference Dye, 0.5 µL of primer 759R, 0.5 µL of primer 760F, and 2 µL template DNA. The qPCR protocol was as follows: pre-denaturation at 95℃ for 5 min, followed by 40 cycles of denaturing at 95℃ for 30 s, annealing at 62℃ for 30 s, and extension at 72℃ for 30 s. All analyses were performed in triplicate, including negative controls with ddH_2_O. The gene copy numbers of each sample were calculated through a regression equation relating to the cycle threshold (Ct) value to the known number of copies in the standards as our previous work [43].

### Bacterial isolation and taxonomic identification

Root samples collected from the Ca800 treatment were used for bacterial isolation, as the Ca-enriched taxa (ASVs) were identical to those found in the Ca300 and Ca400 treatments, but their relative abundances were highest in the Ca800 treatment. The isolation method and culture medium were carried out as follows: First, 1 g of tomato roots were surface-sterilized with 75% ethanol for 30 s, 5% sodium hypochlorite solution for 2 min, and then washed with sterile water for three times. Second, the roots were cut into small pieces and placed in a mortar. Nine mL of PBS buffer was added, and the root cuts were ground thoroughly. The homogenate was filtered with 4 layers of gauze. All operations were performed under a sterile environment. Third, supernatants containing microorganisms were diluted to a series from 10 to 10,000 times with PBS buffer. Then 100 µL of each serial dilution was inoculated onto three agar plates with different nutrients to obtain various types of bacteria. The agar plates were 0.25 × R2A (yeast extract 0.125 g L^−1^, tryptone 0.125 g L^−1^, casamino acid 0.125 g L^−1^, glucose 0.125 g L^−1^, soluble starch 0.125 g L^−1^, sodium pyruvate 0.075 g L^−1^, K_2_HPO_4_ 0.075 g L^−1^, MgSO_4_·7H_2_O 0.0125 g L^−1^, agar 15 g L^−1^, pH 7.2 ± 0.2), 0.1 × TSA (tryptone 1.5 g L^−1^, soy peptone 0.5 g L^−1^, NaCl 0.5 g L^−1^, agar 15 g L^−1^, pH 7.0), and 0.1 × GS (soluble starch 2 g L^−1^, KNO_3_ 0.1 g L^−1^, K_2_HPO_4_ 0.05 g L^−1^, MgSO_4_·7H_2_O 0.05 g L^−1^, NaCl 0.05 g L^−1^, FeSO_4_·7H_2_O 0.001 g L^−1^, agar 15 g L^−1^, pH 7.2). All the plates were cultured at 28℃ for 2–6 days. A limited number of morphologically distinct colonies emerged across the three solid medium. These phenotypically diverse isolates were subsequently re-streaked for purification on fresh agar plates, and individual colonies were cryopreserved in 40% (v/v) glycerol stocks at − 20℃ for long-term storage.

A loop of pure cultures was suspended in 100 µL ddH_2_O, heated at 95℃ for 35 min, and then ice-cooled for 2 min. DNA templates were obtained by centrifugation at 12,000 rpm for 2 min. The primers GM3F (5’-AGAGTTTGATCATGGC-3’) and GM4R (5’-TACCTTGTTACGACTT-3’) were used to amplify the 16S rRNA gene of bacterial samples. The PCR reaction was performed using a 50-µL system consisting of 5 µL 10 × buffer, 4 µL dNTP, 0.5 µL rTaq (Takara), 1 µL primer GM3F (10 µM), 1 µL primer GM4R (10 µM), 2 µL template DNA, and 36.5 µL ddH_2_O. PCR amplification was carried out as follows: pre-denaturation at 94℃ for 5 min, followed by 30 cycles of denaturing at 94℃ for 45 s, annealing at 55℃ for 35 s and extension at 72℃ for 45 s, with a final elongation at 72℃ for 10 min. The PCR products were detected on an 1% agarose gel and sequenced at the Institute of Crop Sciences, Chinese Academy of Agricultural Sciences. Taxonomic annotation was performed against NCBI databases.

### Biocontrol activity of isolated strains

The biocontrol activity of the isolated bacterial strains was estimated by testing their inhibitory effects on the pathogenic strain P380. Briefly, 10 mL of an overnight culture of P380 was added to 250 mL of sterile NA medium maintained at 55℃. After thorough mixing by shaking, the medium was poured into individual Petri dishes and allowed to solidify. Subsequently, 3 µL of overnight culture from each bacterial isolate was spotted at the center of the cooled, solidified plates. Each inhibition assay was performed in triplicate. All plates were incubated at 28℃ for 2 days, after which the presence of inhibition zones was examined. Four strains (i.e., *Dyella japonica*, *Rhodanobacter glycinis*, *Paenibacillus polymyxa*, and *Pseudomonas aeruginosa*) were identified as antagonistic to P380.

### Bacterial genome sequencing and gene function prediction

Whole-genome sequencing of the four antagonistic strains (*Dyella japonica*, *Rhodanobacter glycinis*, *Paenibacillus polymyxa*, and *Pseudomonas aeruginosa*) was performed using the Illumina Hiseq2500 PE150 platform (Novogene Co., Ltd.). Raw reads containing low-quality bases (mass value ≤ 20) were removed to ensure data quality. Genome assembly was conducted using SOAP, Spades, and Abyss software, followed by integration of the assembled contigs with CISA software. Gene prediction was performed using GeneMarkS software (http://topaz.gatech.edu/), and functional annotation of the predicted genes was conducted using the KEGG database.

### Growth of antagonistic bacteria in response to Ca and Ca-enriched root metabolites

Based on the metabolic changes observed in tomato roots following Ca addition, SA, sucrose, and trehalose were selected for further verification. The effects of Ca and Ca-enriched root metabolites on the growth of strain P380 and four antagonistic strains were evaluated in 96-well plates. For Ca treatments, two concentrations of CaCl_2_ (1.385 and 2.769 g L^−1^) were added to NB liquid medium. For metabolite treatments, two concentrations of SA (0.125 and 0.25 mM) and two concentrations of sucrose and trehalose (2.5 and 5 mM) were added to 1/10 LB liquid medium. In all assays, 198 µL of respective medium was inoculated with 2 µL of fresh overnight bacterial culture, and cultures were incubated at 28℃ with shaking at 150 rpm. Bacterial growth was measured at 4, 12, and 24 h by recording optical density at 600 nm (OD_600_) using a multifunctional microplate reader (Thermo Fisher Scientific, Germany). Each assay was conducted in quadruplicate.

### Construction of synthetic community (SynCom)

We constructed a bacterial SynCom consisting of four root-derived strains identified in this study: *Dyella japonica*, *Rhodanobacter glycinis*, *Paenibacillus polymyxa*, and *Pseudomonas aeruginosa*. These strains were selected for SynCom construction based on they: (1) were ranked as significant predictors of pathogen suppression in the Random Forest analysis, (2) exhibited in vitro antagonistic activity against *R. solanacearum*, (3) were promoted by Ca supplementation, (4) grew fast than *R. solanacearum* in response to plant metabolites enhanced by Ca, and (5) contained multiple plant-growth promoting traits in genomic annotation (e.g., phosphorus and iron mobilization, IAA, and siderophores generation). Each strain was cultured in LB liquid medium, and bacterial suspensions were prepared by resuspending harvested cell pellets in sterile ddH_2_O, adjusting the OD_600_ to 1. The four suspensions were then mixed in equal volumes (1:1:1:1) to create the final synthetic bacterial inoculum.

### In vitro interactions among members of SynCom

To explore the interaction among the four members of the SynCom, we performed in vitro pairing interactions (*n* = 4 strains, 10 combinations) on solid plate [44]. Four strains were inoculated in 5 mL LB liquid medium and incubated overnight in a rotary shaker (150 rpm) at 28℃. The optical density of the bacterial cultures was adjusted to 0.1 at 600 nm. Five times 2 µL of these bacterial cultures were inoculated in diagonal row on square petri-dish with LB agar, creating a V-shape of increasingly closer inoculation sites. The plates were incubated for 2 days at 25℃.

### Effect of whole root exudates on growth of *R. solanacearum* and the SynCom members

After 30 days of growth, tomato seedlings were gently uprooted and washed with sterile water to remove all visible soil particles. Cleaned seedlings were transferred to hydroponic containers containing Hoagland nutrient solution under two conditions: (1) 0 mM Ca and (2) 20 mM Ca (as CaCl_2_), with 22 uniformly sized seedlings per treatment. After 5 days of hydroponic growth in a light incubator (28℃, 16/8 h light/dark cycle), seedlings were rinsed thoroughly with sterile distilled water and placed individually into flasks containing 50 mL of deionized water, fully submerging the roots. Flasks were incubated under the same conditions for 48 h to collect root exudates. A total of 1100 mL of exudate per treatment was filtered through 0.22-µm sterile membranes (Millipore), lyophilized, redissolved in 22 mL of sterile water, and stored at − 80℃ until use.

To assess the effects of root exudates on bacterial growth, strain P380 and four strains of SynCom were cultured in 96-well plates containing 100 µL of 1/2 NB liquid medium, 10 µL of fresh overnight liquid bacterial culture, and 30 µL of root exudate solution. Optical density (OD_600_) was measured at 6, 12, 24, and 48 h using a multifunctional microplate reader (Thermo Fisher Scientific, Germany). Each treatment was performed in quadruplicate. For co-culture assays, SynCom strains were mixed in equal volumes (1:1:1:1) to prepare a combined suspension (OD_600_ = 0.1 ± 0.05). The system consisted of 100 µL of 1/2 NB liquid medium, 10 µL of *R. solanacearum* suspension, 10 µL of SynCom suspension, and 30 µL of a root exudate solution. After incubation for 6, 12, 24, and 48 h, 2 µL of culture was collected for qPCR to quantify *R. solanacearum* using strain-specific primers (759R and 760F).

### Biocontrol ability of SynCom

Tomato seeds were germinated on a plate for 2 days at 28℃ and transferred into 80 g of sterilized nursery soil. The following treatments were set up: (1) no Ca fertilizer and no SynCom (Ca0), (2) no Ca but with SynCom, (3) 100 mg Ca kg^-1^ soil, no SynCom (Ca100), and (4) 100 mg Ca kg^-1^ soil with SynCom. Ca supplement was applied to the soil 1 week after the tomatoes sprout. When the tomatoes reached the triloba stage, 10-mL suspensions of *R. solanacearum* were inoculated into all treated soils as described above, and after a week, 10-mL suspensions of SynCom were poured into the soil. Each treatment included nine tomato plants and three replicates. Disease occurrence was monitored as mentioned previously.

### Statistical analysis

One-way analysis of variance was used to analyze the data of chemical properties of soil, plant hormones, activities of enzyme, density of *R. solanacearum*, the microbial diversity, and the incidence of bacterial wilt in the pot experiments involving the combined treatment of SynCom and Ca. Statistical analyses were performed using R software (version 4.4.2) with the following packages: The ANOVA was performed with the *aov* function in the “stats” package. Multiple comparisons were carried out with the *LSD.test* function in “agricolae” package. Principal coordinates analyses (PCoA) were performed using the *pcoa* function in the “vegan” package and the Shannon index was calculated by the *diversity* function. The Mantel rest was performed using the *mantel_test* function in the “ggcor” package and linear regression model was performed using the *stat_poly_eq* function in the “ggpmisc” package, which were used to evaluate the effects of Ca levels on microbial composition in roots. The random forest analysis was performed using the *randomForest* function in the “randomForest” package. The plant-beneficial bacteria (PBB) database was used to identify bacterial genera with potential plant-beneficial functions [45]. Differential analysis of root metabolites was conducted using the *glmLRT* function in the “edgeR” package, and results were visualized with volcano plots to identify significantly altered metabolites (log_2_FoldChange > 1, *p* < 0.01). The phylogenetic tree was constructed with “IQ-TREE” and then annotated and shown using the iTOL (https://itol.embl.de/).

## Results

### Ca impacted tomato growth in a dose-dependent manner

To explore the effect of Ca on tomato growth, we first conducted a preliminary gradient experiment using a wide range of Ca concentrations in the absence of *R. solanacearum*. The results showed that plant height gradually increased from Ca0 up to approximately Ca400, while from Ca500 onwards, plant height progressively declined with increasing Ca levels (Fig. S1). Based on these findings, representative Ca concentrations were selected for a subsequent experiment involving *R. solanacearum* inoculation. The results consistently demonstrated that Ca supplementation within an optimal range promoted tomato plant growth, whereas excessive Ca levels inhibited it (Fig. S2). On day 20 after inoculation, the highest values were recorded at a Ca supplementation level of 400 mg kg^-1^ soil, with plant height reaching 74 cm (Fig. S2a) and fresh weight reaching 59.75 g (Fig. S2b), which were the greatest among all treatments. However, at 800 mg Ca kg^-1^ soil, tomato growth was significantly suppressed, possibly due to high Ca^2+^ concentrations impairing root development. By day 30 after inoculation, no significant differences in either plant height or fresh weight were observed across treatments. Additionally, the total Ca content in the plants did not differ significantly across treatments (Table. S1). In contrast, total nitrogen content increased with higher Ca supplementation levels, suggesting that Ca enhanced the absorption and utilization of nitrogen in tomato plants (Table. S1).

### High doses of Ca reduced tomato wilt incidence

**Fig. 2:**
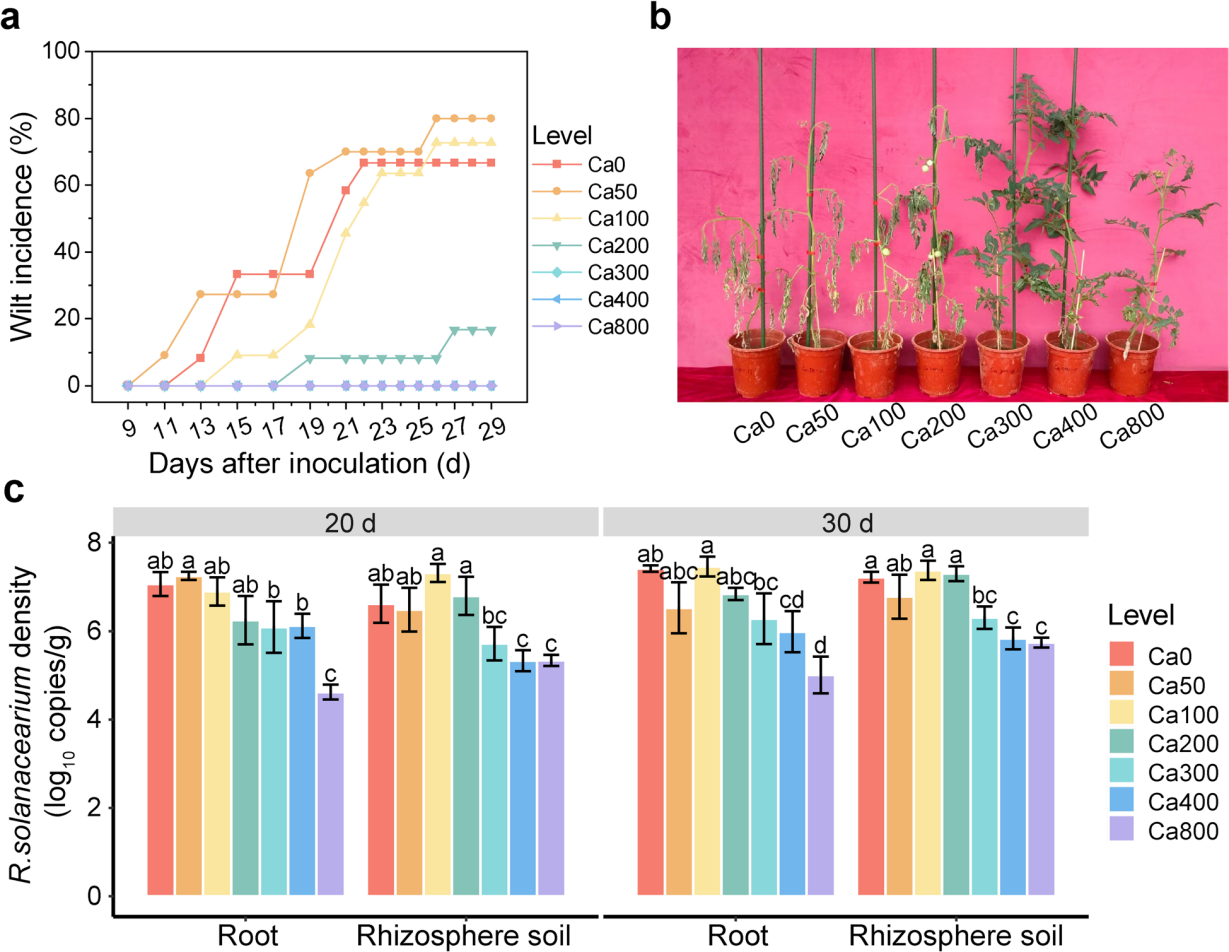
Calcium suppresses the occurrence of bacterial wilt in tomato plants in a dose-dependent manner. **a** The wilt incidence in tomato plants under seven different doses of Ca. **b** Representative tomato plants under different Ca treatments at day 30 after inoculation. **c** Inhibitory effects of increasing Ca levels on the density of *R.* *solanacearum* in roots and rhizosphere soil at two sampling time points. Note: Data are presented as means ± standard error of four replicates (*n* = 4). Different letters indicate significant differences among different Ca treatments according to *LSD* test (*p* < 0.05)

We monitored disease symptoms in tomato plants following* R. solanacearum* inoculation under different Ca treatments. No symptoms were observed in any treatment during the first 9 days post-inoculation (Fig. 2a). Starting on day 9, some plants began to show complete wilting, with no recovery observed despite shading and watering, indicating successful infection by *R. solanacearum*. From day 9 to day 17, bacterial wilt symptoms appeared exclusively in the Ca0 and low-Ca treatments (50 and 100 mg Ca kg^-1^ soil), with slow disease progression (incidence rate: 0–30%). From day 19 onward, the disease incidence in the Ca0, Ca50, and Ca100 treatments increased sharply, reaching peak incidences of 66.67%, 80%, and 72.73%, respectively (Fig. 2a). In contrast, the Ca200 treatment began to exhibit wilting symptoms on day 19 post-inoculation, with an incidence rate of 16.67% by day 30 after inoculation. Notably, no wilting symptoms were observed throughout the entire trial in the Ca300, Ca400, and Ca800 treatments (Fig. 2b).

Real-time PCR analysis further revealed differences in the density of *R. solanacearum* in roots and rhizosphere soil under various Ca treatments (Fig. 2c). At both sampling time points, the density of *R. solanacearum* in the roots and rhizosphere soil showed no significant change in the Ca0, Ca50, Ca100, and Ca200 treatments. However, a significant reduction (one-way ANOVA: *p* < 0.05) in the density of *R. solanacearum* was observed when the Ca concentration reached 300 mg kg^-1^ soil or higher. For instance, on day 20 post-inoculation, the density of *R. solanacearum* in roots under the Ca800 treatment decreased by approximately 34.65% (the copy number of 16S genes decreased from 10^7.07^ to 10^4.62^). In the rhizosphere soil, the density decreased by 19.34% (the copy number of 16S genes decreased from 10^6.62^ to 10^5.34^) compared to the Ca0 treatment. This reduction may be partially attributed to the direct inhibitory effect of Ca on *R. solanacearum*. Interestingly, the density of *R. solanacearum* in roots under Ca800 was consistently lower than that in rhizosphere soil at both sampling time points, suggesting a root-mediated bacteriostatic effect. These results underscore the importance of further exploring the root-mediated mechanisms of disease inhibition under Ca regulation.

### Ca improved tomato immunity

**Fig. 3:**
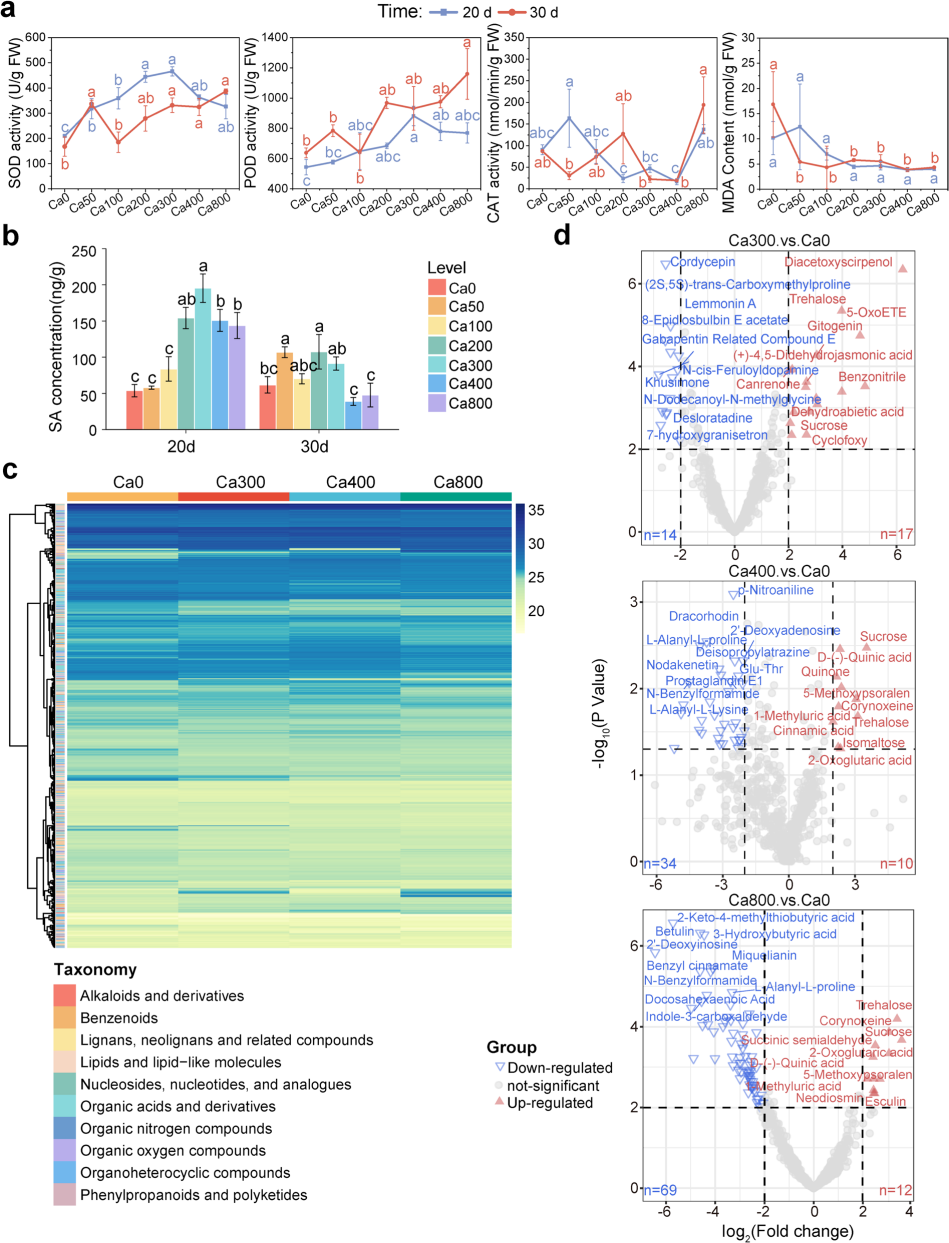
Calcium affects tomato root physiology associated with disease resistance. **a** Impacts of Ca on the enzyme activities (SOD, POD, CAT) and MDA content in tomato roots. **b** Ca affects the concentrations of SA in tomato roots in a dose- and time-dependent manner. **c** Metabolite profiles in tomato roots with or without Ca supplementation. Metabolites were categorized based on their chemical properties and visualized as heatmap. **d** Volcano diagrams show the statistically significantly upregulated, downregulated, and unchanged metabolites in Ca300-treated, Ca400-treated, and Ca800-treated root samples compared to Ca0 (*p* < 0.01). Note: Data are presented as mean ± standard error (*n* = 4). Different letters indicate significant differences among treatments according to *LSD* test (*p* < 0.05) in panels **a** and **b**

To investigate the Ca-mediated defense response of tomato plants against *R. solanacearum* under varying Ca levels, we assessed several physiological indicators in tomato roots, including antioxidant enzyme activities, oxidative damage markers, and hormone levels (Fig. 3). By day 20 after inoculation, SOD activity increased across all Ca-treated samples, with the Ca300 treatment showing a 2.5-fold increase compared to Ca0 (Fig. 3a). By day 30 after inoculation, SOD activity generally continued to rise with increasing Ca concentrations, except in the Ca50 treatment, where the increase was less pronounced than that observed on day 20 (Fig. 3a). POD activity showed a consistent upward trend at both time points, regardless of Ca doses (Fig. 3a). In contrast, CAT activity remained unchanged under all treatments at both sampling time points (Fig. 3a). The content of MDA decreased with increasing Ca levels at both time points (Fig. 3a), indicating that higher Ca concentrations alleviate oxidative damage and enhance cellular stress tolerance.

Ca also significantly influenced the concentration of the defense-related plant hormone SA in a dose- and time-dependent manner (Fig. 3b). On day 20 after inoculation, SA levels in roots treated with Ca200 were nearly three times higher than those in the control. The Ca300 treatment resulted in the highest SA concentration (195.37 ng g^−1^) among all treatments. However, by day 30 after inoculation, further increases in Ca did not significantly alter SA levels. To explore the underlying metabolic changes, root metabolite profiles were analyzed on day 30 after inoculation (Figs. 3c and 3d). Heatmaps revealed marked differences in metabolite composition among the Ca0, Ca300, Ca400, and Ca800 treatments (Fig. 3c). A differential analysis (*p* < 0.01) comparing Ca-treated samples to the Ca0 group (Fig. 3d) showed that the abundance of carbohydrates (e.g., sucrose and trehalose) was upregulated in all Ca-treated roots. Organic acids (e.g., D-(-)-quinic acid) were particularly elevated in the Ca400 and Ca800 treatments. Conversely, certain metabolites, such as N-benzylformamide, were reduced in these high-Ca treatments. Notably, the overall metabolic profiles of Ca300, Ca400, and Ca800 were highly similar, indicating that Ca supplementation activated comparable defense-related metabolic pathways across different concentrations.

### Ca shaped rhizosphere and endophytic bacterial communities

**Fig. 4:**
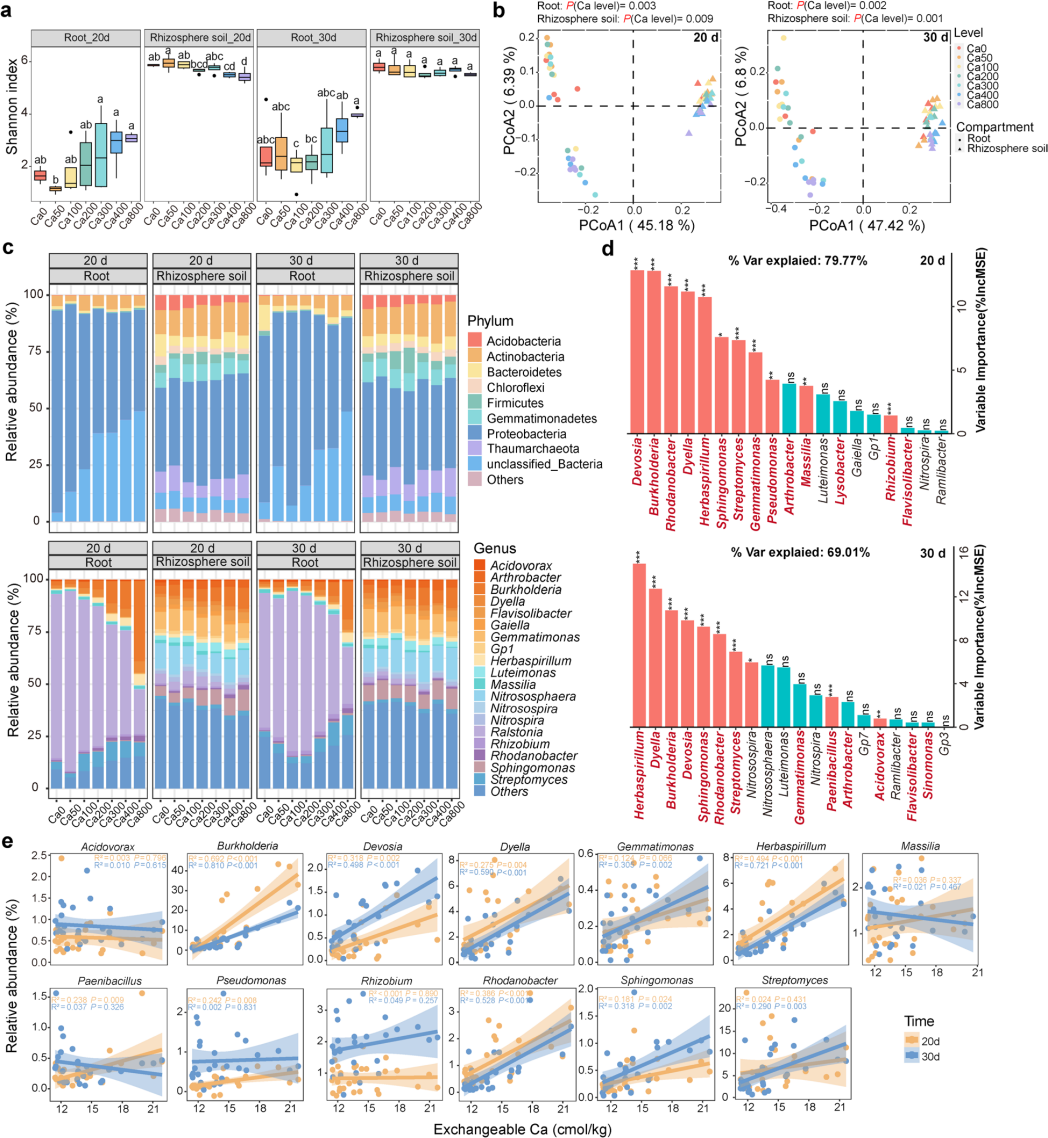
Calcium affects the bacterial communities in tomato roots and rhizosphere soil in a dose-dependent manner. **a** Shannon diversity index of bacterial community in roots and rhizosphere soil at two sampling time points. Note: Different letters indicate significant differences at* p* < 0.05 among different Ca treatments (*n* = 4) according to *LSD* test. **b** Principal coordinate analysis (PCoA) of bacterial communities based on the weighted UniFrac distance at two sampling time points. The Mantel test was used to assess the effect of Ca levels. **c** Stack bar plots depicting the relative abundance of dominant bacterial phyla and genera in root and rhizosphere communities. **d** Important bacterial genera in roots correlated with pathogen density based on the random forest predictive modeling at two sampling time points. Bacterial genera marked in red bold correspond to validated beneficial microbes according to the PBB database. **e** Relationship between Ca application and the relative abundance of beneficial genera by using linear regression analysis

To elucidate the mechanism by which Ca inhibits tomato bacterial wilt, we further examined the effects of Ca application on the bacterial community composition in both roots and rhizosphere soil (Fig. 4). On day 20 after inoculation, bacterial diversity in the roots increased with higher Ca levels, whereas diversity in the rhizosphere soil gradually decreased (Fig. 4a). Despite this decline, rhizosphere soil consistently exhibited higher overall diversity than root samples. By day 30 after inoculation, root diversity trends remained similar to those observed on day 20, while no significant differences in rhizosphere diversity were detected among the different Ca treatments.

Unconstrained principal coordinate analysis (PCoA), based on Bray–Curtis distances, revealed clear separation in bacterial community structures along the first principal coordinate axis for both root and rhizosphere samples at both time points (Fig. 4b), explaining 45.18 and 47.42% of the total variance, respectively. Ca application significantly altered bacterial community composition in roots (Mantel test: *P* = 0.003 on day 20, *P* = 0.002 on day 30) and rhizosphere soil (Mantel test: *P* = 0.009 on day 20, *P* = 0.001 on day 30).

At the bacterial taxonomic level, bacterial composition patterns were largely consistent between day 20 and day 30, but differed notably between root and rhizosphere samples (Fig. 4c). In root samples under the Ca0 treatment, Proteobacteria comprised over 80% relative abundance. As Ca levels increased from 0 to 800 mg kg^-1^ soil, the relative abundance of Proteobacteria in roots declined approximately to 40%, replaced largely by unclassified bacteria. In contrast, the relative abundance of Proteobacteria in rhizosphere samples remained stable at around 40%. At the genus level, *Ralstonia* was over 80% in root samples under Ca0 and Ca50 treatments but declined progressively with increasing Ca levels, dropping to 30–35% in higher-Ca treatments (Fig. 4c). This reduction was accompanied by an increase in genera such as *Burkholderia*, *Dyella*, *Herbaspirillum*, *Rhodanobacter*, and others. In contrast, bacterial compositions in rhizosphere soil were distinct from those in roots and exhibited less pronounced changes in response to Ca treatment.

Statistical analyses of dominant bacterial genera revealed both temporal and spatial variations in microbial composition (Fig. S3). Ca supplementation significantly reshaped the root microbiome, enriching sixteen bacterial genera, including *Arthrobacter*, *Burkholderia*, *Dyella*, *Herbaspirillum*, *Rhodanobacter*, *Paenibacillus*, and *Pseudomonas*. For example, under the Ca800 treatment on day 30 post-inoculation, *Burkholderia* increased by 16.5-fold, *Rhodanobacter* by 10.2-fold, and *Pseudomonas* by 1.54-fold compared to Ca0 treatment (one-way ANOVA: *p* < 0.05) (Fig. S3). A random forest model further identified twenty-six bacterial genera (e.g., *Burkholderia*, *Dyella*, *Rhodanobacter*, *Paenibacillus*, *Pseudomonas*) as potential contributors to reducing the density of *R. solanacearum* in roots (Fig. 4d). Among these, 17 genera (highlighted in bold red in Fig. 4d) were confirmed as beneficial based on the PBB database. To assess the relationship between bacterial enrichment and Ca availability, linear regression analyses were performed between the relative abundance of 13 beneficial genera (identified as significant contributors by random forest modeling) and soil exchangeable Ca content (Fig. 4e). Several genera, including *Burkholderia*, *Devosia*, *Dyella*, *Herbaspirillum*, *Rhodanbacter*, and *Sphingomonas*, exhibited consistent positive correlations with Ca levels at both time points (Fig. 4e). In contrast, *Paenibacillus* and *Pseudomonas* showed transient correlations only on day 20 post-inoculation, while no significant correlations were observed for *Acidovorax*, *Massilia*, or *Rhizobium* (Fig. 4e).

### Key Ca-stimulated bacterial isolates showed biocontrol efficacy and plant growth-promoting activities

**Fig. 5:**
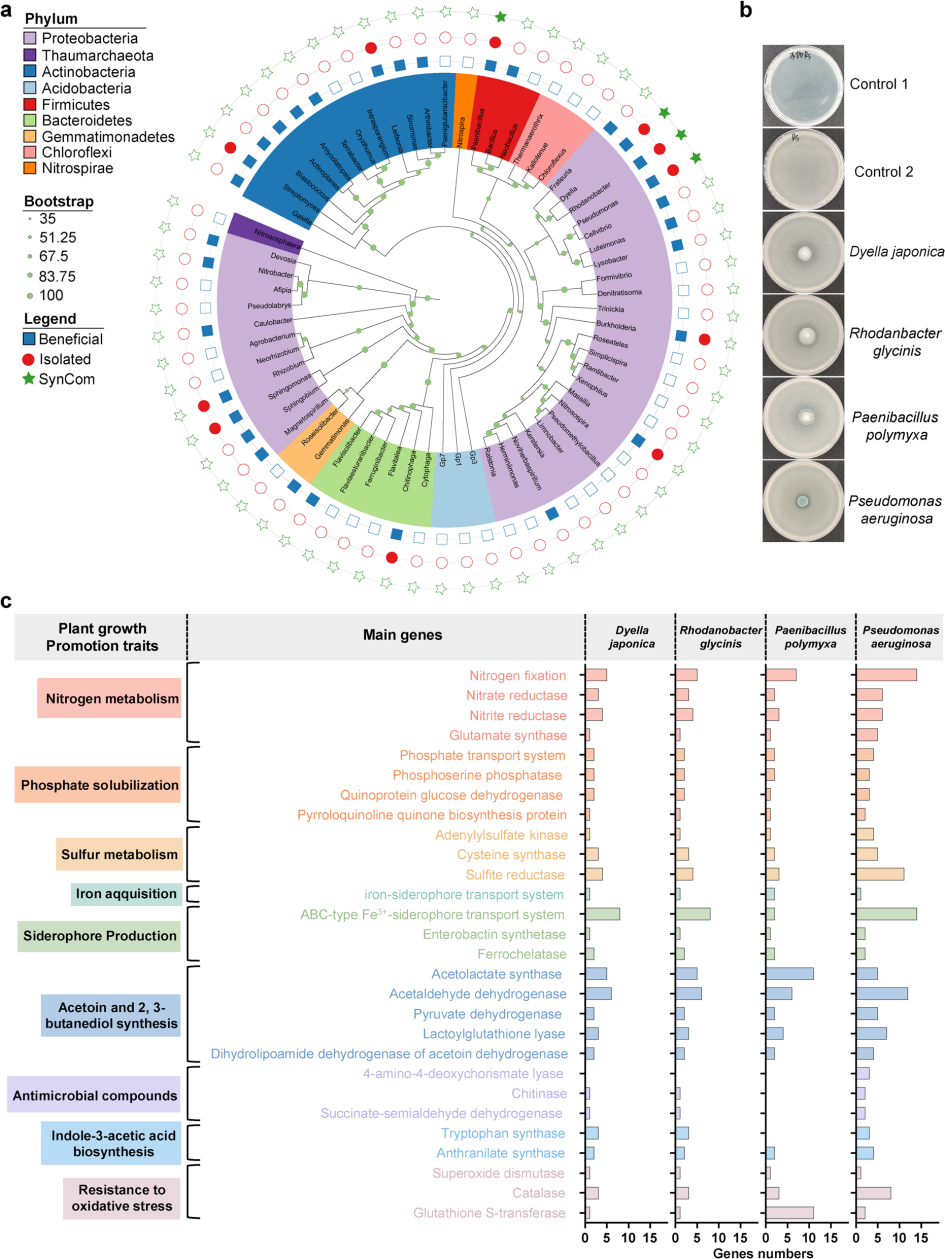
Key Ca-stimulated bacterial isolates showed biocontrol efficacy and plant growth-promoting activities. **a** Phylogenetic tree of bacterial amplicon-sequence-variants (ASVs) with relative abundances greater than 0.1% in tomato roots. The inner ring colors represent the phylum-level classification of each ASV. Labels: “Beneficial” denotes genera identified as beneficial based on the PBB database; “Isolated” refers to genera successfully isolated in this study; and “SynCom” refers to genera included in the synthetic community. **b** Inhibitory effects of four Ca-stimulated bacterial strains isolated in this study on *R. solanacearum* growth, assessed via NA plates assays. **c** Number of functional genes related to plant growth-promoting traits identified in the Ca-stimulated bacterial strains

To visually highlight the key beneficial genera and those we successfully isolated, a phylogenetic tree was constructed (Fig. 5a). Based on microbiome profiling and comparison with the PBB database, several bacterial genera were identified as potentially beneficial for suppressing *R. solanacearum* and promoting tomato health (labeled as “Beneficial” in the legend). Among these, we isolated 11 bacterial genera (labeled as “Isolated”). The inhibitory effects of the isolated strains on *R. solanacearum* were assessed via plate assays. Notably, four genera (*Dyella*, *Rhodanobacter, Paenibacillus*, and *Pseudomonas*) categorized as both “Beneficial” and “Isolated” showed significant inhibitory effects on the growth of *R. solanacearum* (Fig. 5b). The 16S rRNA gene sequences of these four strains matched representative sequences from high-throughput sequencing (Fig. S4), confirming their identities as *Dyella japonica*, *Rhodanobacter glycinis*, *Paenibacillus polymyxa*, and *Pseudomonas aeruginosa*.

To further elucidate the functional potential of these four bacterial strains, we conducted whole-genome sequencing followed by KEGG pathway annotation (Fig. 5c). Genomic analysis revealed these four beneficial strains harbor genes linked to plant growth promotion, including those involved in phosphorus solubilization (e.g., phosphate transport system), siderophore production (e.g., ABC-type Fe^3+^-siderophore transport systems), and oxidative stress resistance (e.g., catalase and glutathione S-transferase). Pairwise interaction assays showed no strong antagonism among these four bacterial strains (Fig. S5). Based on the selection criteria described in the Materials and Methods section, these four strains were subsequently chosen for the construction of a SynCom.

### Ca-enriched whole root exudates and key metabolites inhibited *R. solanacearum* growth and promoted the growth of SynCom members under in vitro conditions

**Fig. 6:**
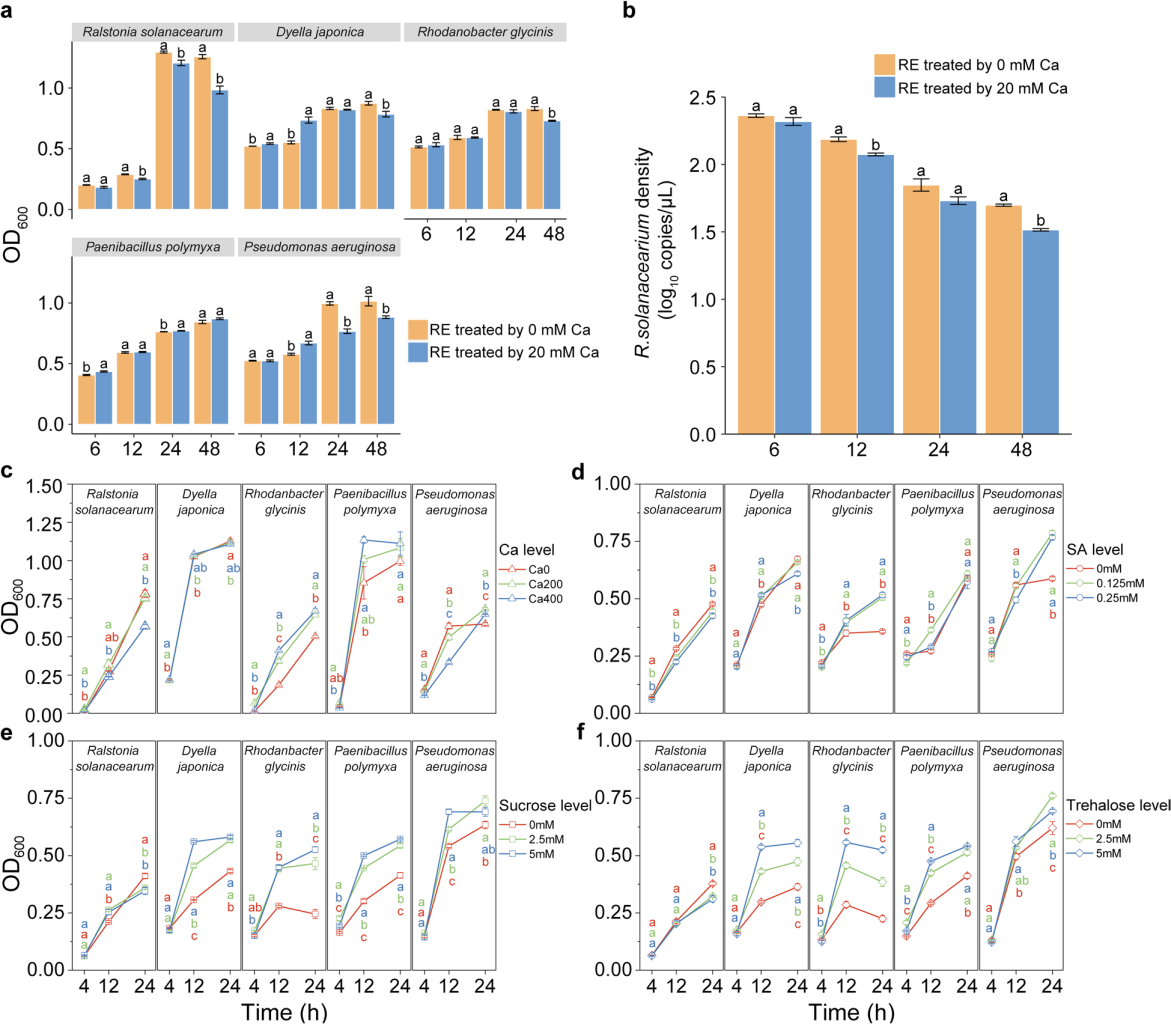
Suppression of *R. solanacearum* by whole root exudates, synthetic community members, calcium, SA, sucrose, and trehalose.** a** Effects of whole root exudates on the growth of *R. solanacearum* and SynCom members. **b** Effects of whole root exudates on the density of *R. solanacearum* during co-culture with SynCom. **c** Effects of varying Ca concentrations on the growth of *R. solanacearum* and SynCom members in liquid NB medium. **d–f** Effects of different concentrations of SA, sucrose, and trehalose on the growth of *R. solanacearum* and SynCom members in liquid LB medium. Growth curves are presented as mean ± SE (*n* = 4). Note: Different letters indicate significant differences at* p* < 0.05 among substrate concentrations according to *LSD* test

To bridge the mechanistic link between Ca application, plant metabolites, pathogen inhibition, and the recruitment of beneficial microbes, we extracted whole root exudates from tomato plants treated with 0 and 20 mM Ca and assessed their effects on *R. solanacearum* and four beneficial bacterial strains. The results revealed that whole root exudates from Ca-treated plants (20 mM) significantly inhibited *R. solanacearum* growth while promoting the growth of beneficial microbes such as *Paenibacillus polymyxa* (Fig. 6a). In a co-culture experiment involving *R. solanacearum* and the SynCom, the addition of whole root exudates from 20 mM Ca-treated plants led to a significant reduction in *R. solanacearum* density, indicating a synergistic inhibitory between the SynCom and Ca-induced whole root exudates (Fig. 6b).

We further investigated the direct effects of Ca by examining the growth responses of *R. solanacearum* and the four beneficial strains under various Ca concentrations. Ca supplementation significantly (one-way ANOVA: *p* < 0.05) inhibited the growth of *R. solanacearum* while enhancing the growth of the four beneficial strains (Fig. 6c). Additionally, higher concentrations of key metabolites (SA, sucrose, and trehalose) inhibited the growth of *R. solanacearum* and simultaneously promoted the proliferation of beneficial microbes such as *Rhodanobacter glycinis* and *Pseudomonas aeruginosa* (Figs. 6d–f). Collectively, these results demonstrate that Ca-induced whole root exudates and root metabolites can differentially modulate pathogenic and beneficial microbial populations, thereby restructuring the root microbiome as part of the plant’s defense strategy against pathogen attack.

### SynCom inhibited *R. solanacearum* and controlled bacterial wilt with reduced Ca application

**Fig. 7:**
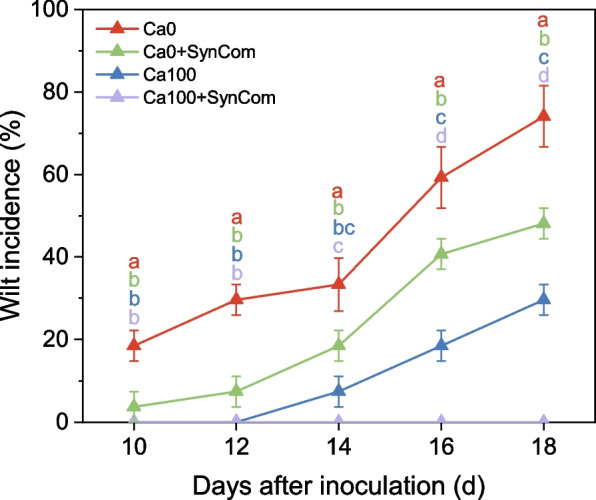
Incidence of bacterial wilt in tomato plants under different treatments with SynCom and Ca in a pot experiment following inoculation with *R. solanacearum*. Four treatments were applied: (1) no Ca fertilizer and no SynCom (Ca0), (2) SynCom only without Ca, (3) 100 mg Ca kg^-1^ soil without SynCom (Ca100), and (4) 100 mg Ca kg^-1^ soil with SynCom. Disease incidence was monitored over time and analyzed using one-way ANOVA. Data are presented as mean ± SE (*n* = 3)

Considering the economic benefits and practical application of Ca in agriculture, we further conducted pot trials to evaluate the combined effects of SynCom and low-dose Ca application on suppression of bacterial wilt (Fig. 7). The results showed that plants in the Ca0 treatment exhibited the earliest disease onset and the highest incidence of bacterial wilt. During day 10 to day 14 after inoculation, there was no significant difference between the application of Ca alone and SynCom alone in controlling the disease. However, starting from day 16 after inoculation, the application Ca alone was more effective than SynCom alone in disease suppression. Importantly, the combination of Ca supplementation with SynCom achieved a synergistic effect, significantly (one-way ANOVA: *p* < 0.05) reducing the incidence of bacterial wilt compared with other treatmentsfrom day 14 after inoculation, with wilt incidence dropping from 74.07 to 0%. This combined approach effectively compensates for the reduced pathogen suppression caused by Ca deficiency.

## Discussion

Targeted selection and utilization of specific microbes to engineer the root microbiome for plant protection holds great promise, but remains technically challenging [46]. In this study, we found that Ca supplementation significantly increased the relative abundance of several bacterial genera in tomato roots, including *Dyella japonica*, *Rhodanobacter glycinis*, *Paenibacillus polymyxa*, and *Pseudomonas aeruginosa* (Fig. S3). These taxa are usually found to be beneficial bacteria that play a direct or indirect role in inhibiting pathogen growth in various plant-pathogen systems [47–49]. For instance, *Rhodanobacter* has been reported to antagonize the root rot fungus *Fusarium solani* and may also participate in nitrogen cycling processes [46]; *Paenibacillus* can produce lipopeptide antibiotics such as polymyxin and fusaridin [50]. In our study, these beneficial microbes not only directly inhibited *R. solanacearum* (Fig. 5b), but also indirectly inhibited its growth by altering the root microhabitat and competing for root exudates (Fig. 6b). These results renew the viewpoint that Ca not only directly protects plants [34, 51], but also enhances their ability to recruit beneficial root microbiome for disease control. This mechanism aligns with prior observations in peanut systems [35], where Ca enriched specific soil microbiota to suppress soil-borne pathogens, suggesting a broad cross-crop pattern of Ca-driven microbiome modulation. Our approach of shaping root microbiome through Ca application is both simple and efficient, unlike previous approaches that take long time or need advanced techniques for resistance breeding, genetic modification or generating non-pathogenic derivatives of pathogen [52, 53]. A recent report showed that phosphorus availability can influence the recruitment of pathogen-suppressive microbes [28]. Taken together, these findings suggest that optimizing mineral nutrient management holds great potential as a sustainable strategy for disease control by strengthening plant-microbe cooperations.

**Fig. 8:**
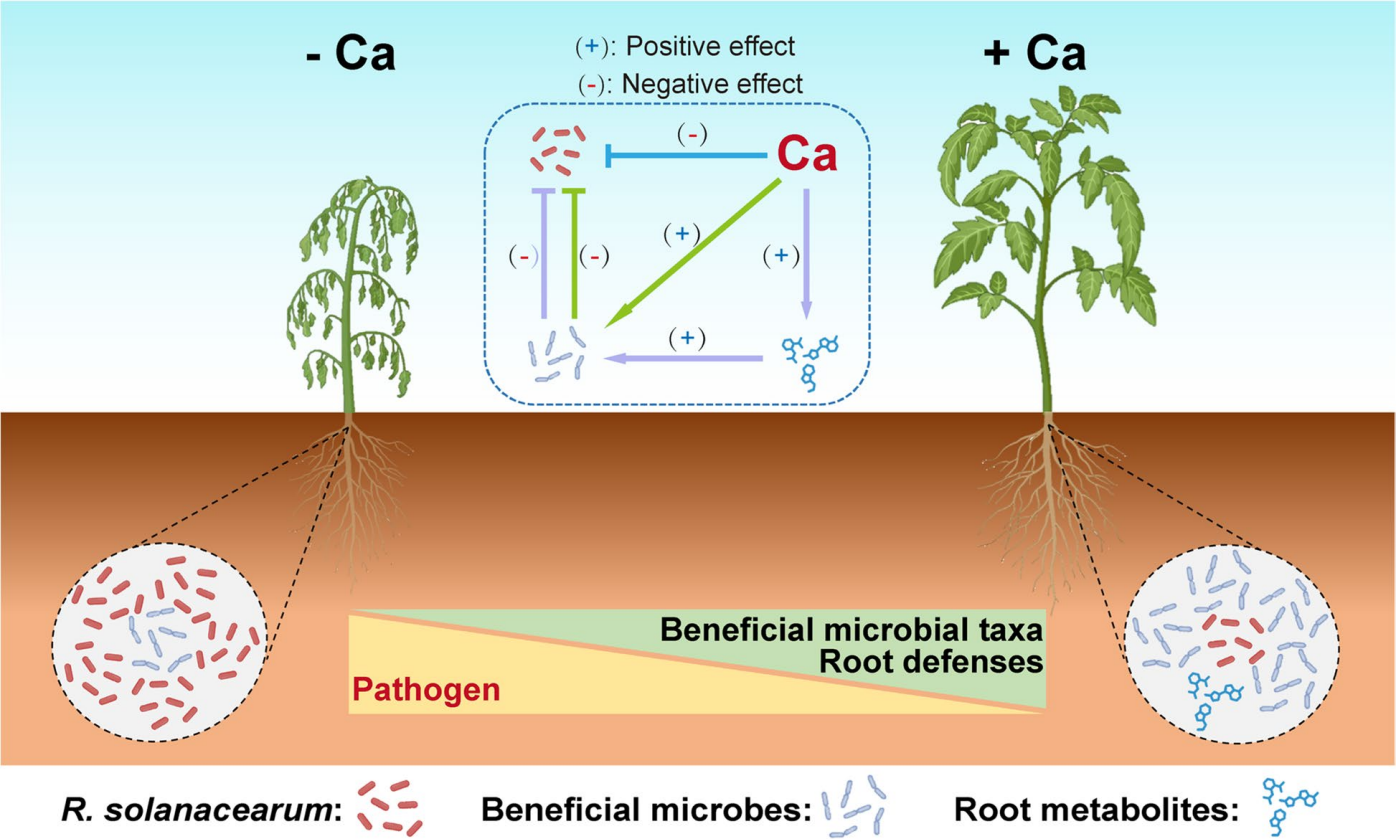
A schematic model illustrating the effects of calcium application on the suppression of tomato bacterial wilt. The model describes three ways by which Ca enhances tomato resistance to *R.* *solanacearum*: (1) direct inhibition, (2) recruitment of beneficial microbial taxa in roots, and (3) induction of root defense responses

To delve into the mechanisms underlying the linkage between Ca and root endophytic microbiomes, we monitored shifts in root metabolism with and without Ca supplementation. We found that the enrichment of specific bacterial taxa, such as *Dyella japonica*, *Rhodanobacter glycinis*, *Paenibacillus polymyxa*, and *Pseudomonas aeruginosa*, was associated with higher SA concentrations in roots. Furthermore, we verified that elevated SA levels promote the growth of these Ca-enriched taxa in vitro (Fig. 6d), consistent with the known linkage between plant immune pathways and root microbiome assembly [23]. Since the activation of plant immunity triggers a cascade of metabolic responses, we assessed root metabolism using untargeted metabolomics and found that several metabolites, such as sucrose and trehalose, were also increased in roots under Ca treatment (Fig. 3d). These metabolites are typically involved in plant immune responses and may contribute to enhanced plant defense [54, 55]. In vitro tests showed that these metabolites also promoted the growth of Ca-stimulated beneficial taxa to varying extents (Fig. 6e and 6f). To directly evaluate the effects of Ca-mediated root exudates, we collected whole root exudates from tomato plants treated with 20 mM Ca, alongside untreated controls (0 mM). In vitro co-culture experiments with *R. solanacearum* and beneficial bacteria demonstrated that whole root exudates from Ca-treated plants significantly reduced the density of *R. solanacearum* compared to control exudates (Fig. 6b), suggesting synergistic interactions between Ca-modified root exudates and probiotic microbiota in pathogen inhibition. Therefore, we believe that microbiome assembly shaped by Ca is collectively facilitated by multiple metabolites involved in plant defense metabolic pathways. Our results did not exclude the direct inhibition of pathogen by Ca, as found in previous studies [34, 51]. Rather, we showed that direct inhibition was quite likely, especially under high Ca conditions as defense responses did not increase further while *R. solanacearum* remained declining. Here, we proposed a model on how Ca supplementation contributes to tomato defense against bacterial wilt caused by *R. solanacearum* (Fig. 8). We emphasize the synergistic effects of Ca supplementation in stimulating both root defenses and beneficial microbial assembly, ultimately enhancing disease suppression.

Alternative strategies to chemical control of plant diseases have long been sought, as pesticide use often causes environmental contamination and induces chemical resistance in pathogens. Root microbiome engineering is increasingly recognized as a promising approach to disease control [56]. Field applications of this approach, such as bioorganic fertilizers containing disease-suppressing microorganisms, often fail to elevate biocontrol agents to sufficient densities needed for effective suppression of soil-borne diseases [57, 58]. Our study demonstrates that high doses of Ca can effectively suppress *R. solanacearum*, but the amount is too large that is costly and may deteriorate soil quality. To address this, we propose a combined strategy involving Ca and a SynCom composed of antagonist strains that thrive under high-Ca conditions (Fig. 7). This approach significantly reduces the requirement of Ca while enhancing disease prevention. It is particularly suitable for soils with low Ca content and a high incidence of *R. solanacearum*. Members of our SynCom also occur frequently in a variety of soil environments, including wheat monoculture systems [59], tea plantations [46], and forest ecosystems [48]. Genome sequencing revealed that these strains possess multiple plant growth-promoting traits (e.g., phosphorus and iron mobilization, IAA, and siderophores generation) (Fig. 5c). Therefore, the Ca-mediated enrichment of these beneficial root-associated microbes may be a broadly applicable strategy across diverse cropping systems and soil types. As the adoption of bioorganic fertilizers with biocontrol microbes continues to increase in cash crop production, the integration of Ca and Ca-preferred biocontrol strains offers a promising enhancement to current formulations. This approach not only provides disease-suppressive agents but also improves plant Ca nutrition and facilitates root microbiome assembly through plant immune priming. Together, these effects represent a novel and synergistic way to coordinating plant health, soil quality, and root microbial communities for effective disease control.

While our study highlights the promising potential of combining Ca supplementation with SynCom to enhance plant resistance to pathogens, several limitations must be acknowledged. First, our experiments were conducted under controlled laboratory conditions, which may not fully capture the complexities of field environments, such as heterogeneous soil types, climatic fluctuations, and dynamic microbial competition. Second, the long-term stability and scalability of Ca and SynCom interactions remain to be validated, particularly across diverse agricultural systems. Third, certain antagonistic interactions among SynCom members may limit its overall efficacy, suggesting that further refinement of the consortium could enhance its disease-suppressing capacity. Future research should prioritize field trials to evaluate the efficacy of these microbial consortia under field conditions. Key areas of investigation include the compatibility of Ca and SynCom with different crop species, optimization of application methods, and sustainability of their interactions over multiple growing seasons. Addressing these challenges is crucial for translating laboratory findings into practical, large-scale agricultural applications.

## Conclusions

In summary, we demonstrated that Ca promotes plant defense via selectively enriching specific beneficial bacterial taxa. The observation from enhanced defense responses to the assembly of suppressive microbiota reveals a new role of Ca in coordinating plant growth and immunity. Synergistic disease suppression by Ca and beneficial microbiota presents a promising strategy for enhancing bioorganic fertilizers through the combined stimulation of plant immunity booster and its compatible biocontrol microbes.

## Data Availability

Raw amplicon sequencing data was deposited in the National Center for Biotechnology Information (NCBI) Sequence Read Archive (SRA) under the project number PRJNA1125171. Raw genome sequences of four bacterial strains in this study can be found under the following accession numbers: *Dyella japonica* (SRR33220703), *Rhodanobacter glycinis* (SRR33220740), *Paenibacillus polymyxa* (SRR33221449), and *Pseudomonas aeruginosa* (SRR33221452).
